# State-Specific Healthy Life Expectancy at Age 65 Years — United States, 2007–2009

**Published:** 2013-07-19

**Authors:** Man-Huei Chang, Heba Athar, Paula W. Yoon, Michael T. Molla, Benedict I. Truman, Ramal Moonesinghe

**Affiliations:** Epidemiology and Analysis Program Office; National Center for Health Statistics; National Center for HIV/AIDS, Viral Hepatitis, STD, and TB Prevention; Office of Minority Health and Health Equity, CDC

Healthy life expectancy (HLE) is a population health measure that combines mortality data with morbidity or health status data to estimate expected years of life in good health for persons at a given age. HLE accounts for quantity and quality of life and can be used to describe and monitor the health status of populations. HLE estimates for countries have been used for predicting future health service needs, evaluating health programs, and identifying trends and inequalities (1), but to date, few studies have reported HLE at the state level for the United States (2). To determine state-level estimates, CDC used data from the National Vital Statistics Systems (NVSS), U.S. Census Bureau, and Behavioral Risk Factor Surveillance System (BRFSS) to calculate HLEs for persons aged 65 years, by sex and race, for each of the 50 states and the District of Columbia (DC). Those calculations indicate that, during 2007–2009, females had a greater HLE than males at age 65 years in every state and DC. HLE was greater for whites than for blacks in all states from which sufficient data were available and DC, except in Nevada and New Mexico. These results can be used as a baseline for states to monitor changes in the HLE of persons aged 65 years as they age and identify health disparities among subpopulations.

State-specific HLE estimates were calculated from three data sources: 1) 2007–2009 state-specific, individual-level multiple cause mortality data from NVSS; 2) 2007–2009 bridged-race, mid-year population estimates from the U.S. Census Bureau; and 3) 2007–2009 self-reported health status data from BRFSS, a state-based, telephone survey of noninstitutionalized U.S. civilian adults aged ≥18 years administered in all states and selected territories.* The BRFSS question used to assess health status was “Would you say that in general your health is excellent, very good, good, fair, or poor?” For this study, participant responses of “fair or poor” were categorized as “unhealthy” and “excellent, very good, or good” as “healthy.” During 2007–2009, the BRFSS median response rate† for states ranged from 50.6% to 53.3% (3).

Life expectancy (LE) (i.e., expected years of life at a given age) is the average remaining years of life a person can expect to live on the basis of the current mortality rates for the population. HLE estimates the equivalent healthy years that a person can expect to live on the basis of the current mortality rates and prevalence distribution of health status in the population. An abridged life table method was used to estimate LE using data by 5-year age intervals (4). State-specific HLE estimates were calculated from the LE estimates and the self-reported health status data from the BRFSS.

To estimate LE, age-specific death rates were calculated using the mid-year U.S. Census population and the number of deaths in the NVSS. Age-specific death rates were used to estimate the number of survivors, the total number of person-years lived within each age interval, and the average expected years of life remaining per person at the beginning of each age interval. To estimate HLE at a given age, the prevalence of being healthy at the beginning of the age interval and the total number of person-years lived by a cohort in that age interval were calculated. The products for each age interval and for all subsequent age intervals were summed to obtain the total number of years lived in healthy state at a given age. This sum was then divided by the number of persons alive at each age interval.§

HLE estimates were calculated for persons aged 65 years, by sex (male and female) and race (white and black) for each of the 50 states and DC. States with small numbers of deaths (<700 total deaths in the period studied) in specific demographic categories were excluded from the analysis (5). HLE estimates for Hispanics, Asians, and American Indians/Alaska Natives were not presented because sufficient reliable data were not available at the state level. State estimates for HLE as a percent of LE for each age and race subpopulation were calculated. Statistical software was used to account for the complex BRFSS sampling. To assess disparities, differences in HLE were measured between subpopulations. The statistical significance of the differences was assessed using the two-tailed z-statistic and p<0.05.

For both sexes, estimated HLE generally was less in the South than elsewhere in the United States (Figure 1). HLE for males at age 65 years varied from a low of 10.1 years in Mississippi to a high of 15.0 years in Hawaii (Table). HLE for females at age 65 years varied from a low of 11.4 years in Mississippi to a high of 17.3 years in Hawaii. HLE was greater for females than for males in all states, with the difference ranging from 0.7 years in Louisiana to 3.1 years in North Dakota and South Dakota.

By race, HLE estimates for whites were lowest among southern states (Figure 2). For blacks, HLE estimates were comparatively low throughout the United States, except for a few southwestern states. For whites aged 65 years, HLE varied from a low of 11.0 years in West Virginia to a high of 18.8 years in DC. For blacks, data for 11 states were not reported because the total number of deaths during 2007–2009 for the black population in those states was <700. For the remaining states, HLE for blacks aged 65 years varied from a low of 7.1 years in Iowa to a high of 15.1 years in New Mexico. HLE was greater for whites than for blacks in most states, except in Nevada and New Mexico where, respectively, HLE for whites was 0.4 and 0.8 years less than for blacks. The largest difference in HLE between whites and blacks was 7.8 years in Iowa.

For the total population at age 65 years, HLE was lowest among southern states (Figure 3). For all persons at age 65 years, the highest HLE was observed in Hawaii (16.2 years) and the lowest was in Mississippi (10.8 years). During 2007–2009, HLE as a percentage of LE for persons at age 65 years for the total U.S. population ranged from a low of 61.5% in Mississippi to a high of 78.2% in Vermont (Table). Conversely, the number of remaining years in fair or poor health for persons aged 65 years was 6.7 out of 17.5 years of LE for those living in Mississippi and 4.2 years out of 19.4 years for those living in Vermont.

## Editorial Note

HLE estimates in this report identified disparities by sex, race, and state among persons aged 65 years. During 2007–2009, females had a greater HLE than males at age 65 years in every state and DC. HLE was greater for whites than for blacks in all states for which sufficient data were available and DC, except for a difference of <1 year that was observed in Nevada and New Mexico. In general, at age 65 years, HLEs within individual states varied up to 3 years by sex and up to 8 years by race. HLEs for all persons aged 65 years varied between states by 6 years.

Over the past century in the United States, a general decline in death rates has resulted in a corresponding increase in LE. Because differences in HLE by demographics might result from variations in morbidity or mortality, examining HLE as a percentage of LE reveals populations that might be enduring illness or disability for more years. Although HLE measures do not identify the reasons for poor health or shorter lives, they provide a snapshot of the health status of a population. From this measure it is not possible to determine why some states have higher HLE than others. Many factors influence a person’s health status as they age, including 1) safe and healthy living environments, 2) healthy behaviors (e.g., exercise and not smoking), 3) getting the recommended clinical preventive services (e.g., vaccines, cancer screenings, and blood pressure checks), and 4) having access to good quality health care when it is needed.

The findings in this report are subject to at least five limitations. First, BRFSS includes a self-assessed health status question, which might be influenced by age, sex, race/ethnicity, culture, and several social and behavior factors, resulting in rankings of health status that might be assessed inconsistently across demographic groups. However, self-reported health status questions, as used in BRFSS, have been shown to be a good predictor of future disability, hospitalization, and mortality (7–8). Second, possible misclassification of demographic information on the death certificate and misclassification because of the bridging procedure used to categorize persons of multiple race in the census data might have occurred. Third, the BRFSS median response rates in the low 50% raise the possibility of response bias. Fourth, BRFSS is a telephone interview-based survey that did not include persons without access to a landline telephone in its 2007–2009 surveys. Finally, state-specific HLE estimates might not be precise for small groups (especially blacks) by age and sex because of small BRFSS samples and low death counts in some states.

HLE measures reflect current mortality rates and health status for various populations and suggest the long-range implications of the prevailing age-specific death and illness rates. HLE is a relatively simple measure that can be readily used by public health officials, health-care providers, and policy makers to understand the health status of a population. The results presented in this study can be used as a baseline for states to monitor the HLE of persons aged 65 years as they age, identify health disparities among subpopulations, and target resources to improve population health.

What is already known on this topic?

Healthy life expectancy (HLE) is a population health measure that combines age-specific mortality with morbidity or health status to estimate expected years of life in good health for persons at a given age. HLE reflects both quality and quantity of life and might be useful in describing and monitoring the health status of a population. Although national HLE estimates for the U.S. population have been reported previously, limited data have been available on estimates at the state level and by demographic characteristics.

What is added by this report?

In this report, differences in HLE were reported at the state level for adults aged 65 years based on self-reported health in the 2007–2009 Behavioral Risk Factor Surveillance System survey, National Vital Statistics Systems mortality data, and corresponding U.S. Census population estimates. The HLE estimates identify disparities in health status by sex, race, and state. Overall, at age 65 years, females had a greater HLE than males and whites had a greater HLE than blacks in all states with sufficient data and the District of Columbia, except in Nevada and New Mexico.

What are the implications for public health practice?

HLE is a relatively simple measure that can be readily used by public health officials, health-care providers, and policy makers to monitor trends in the health of populations and identify health inequalities. The results presented in this study can be used as a baseline for states to monitor changes in the HLE of persons aged 65 years as they age and to identify health disparities among subpopulations by state.

## Figures and Tables

**FIGURE 1:**
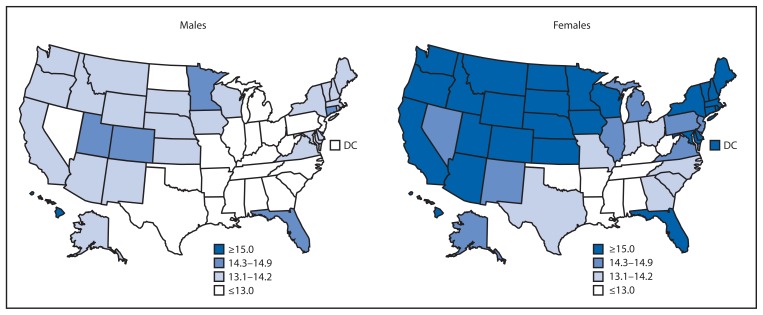
State-specific healthy life expectancy in years at age 65 years, by sex — United States, 2007–2009

**FIGURE 2:**
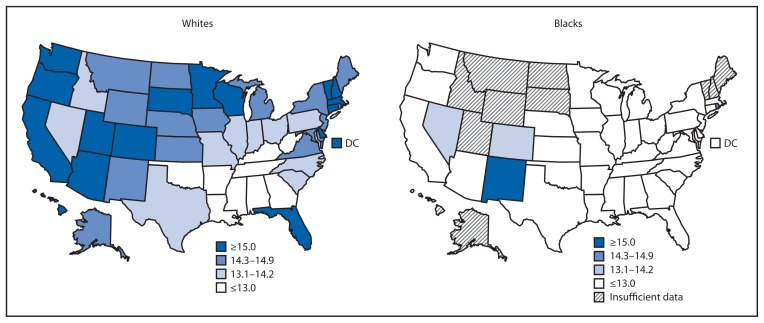
State-specific healthy life expectancy in years at age 65 years, by race — United States, 2007–2009^*^ ^*^ Data for 11 states were not reported because the total number of deaths from 2007 to 2009 for the black population in those states was <700: Alaska, Hawaii, Idaho, Maine, Montana, New Hampshire, North Dakota, South Dakota, Utah, Vermont, and Wyoming.

**FIGURE 3:**
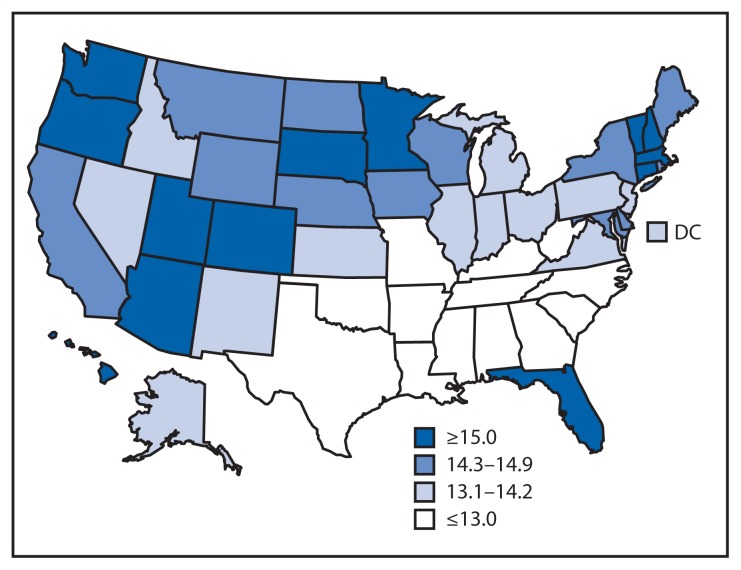
State-specific healthy life expectancy in years at age 65 years —United States, 2007–2009

**TABLE:** State-specific years of life expectancy (LE) and healthy life expectancy (HLE), and percentage of remaining years calculated as healthy (HLE/LE) for persons aged 65 years, by sex and race — United States, 2007–2009

State	All races			Sex						Race					
		
	Both sexes			Male			Female			White			Black		
				
	LE	HLE	(%*)	LE	HLE†	(%)	LE	HLE†	(%)	LE	HLE§	(%)	LE	HLE§	(%)
Alabama	17.6	11.1	(63.0)	16.1	10.3	(64.2)	18.9	11.7	(62.1)	17.8	11.7	(65.7)	16.8	8.4	(50.1)
Alaska	19.1	14.1	(73.9)	18.0	13.3	(73.7)	20.2	14.9	(73.5)	19.5	14.7	(75.7)	—¶	—	—
Arizona	20.2	15.0	(74.6)	18.9	13.7	(72.4)	21.3	16.3	(76.4)	20.1	15.2	(75.4)	19.4	12.9	(66.4)
Arkansas	18.1	12.2	(67.4)	16.6	11.6	(69.5)	19.3	12.7	(65.8)	18.2	12.4	(68.1)	16.7	9.4	(56.1)
California	20.3	14.7	(72.6)	18.9	13.8	(73.0)	21.5	15.5	(72.3)	20.0	15.0	(74.9)	18.2	11.5	(63.4)
Colorado	19.8	15.3	(77.4)	18.5	14.3	(77.4)	20.9	16.2	(77.4)	19.7	16.4	(83.1)	19.2	14.0	(72.6)
Connecticut	20.2	15.7	(77.5)	18.7	14.3	(76.5)	21.3	16.7	(78.3)	20.2	15.8	(78.3)	20.1	12.0	(59.6)
Delaware	19.2	14.7	(76.8)	17.9	13.5	(75.3)	20.2	15.7	(77.8)	19.2	15.0	(78.1)	18.5	12.2	(65.6)
District of Columbia	19.0	14.1	(74.4)	17.1	13.0	(76.2)	20.5	15.0	(73.4)	21.7	18.8	(86.8)	17.6	11.7	(66.5)
Florida	20.4	15.4	(75.3)	19.0	14.3	(75.3)	21.7	16.4	(75.3)	20.5	15.9	(77.3)	19.1	11.7	(61.5)
Georgia	18.2	12.4	(68.4)	16.7	11.6	(69.3)	19.4	13.1	(67.6)	18.3	13.0	(70.8)	17.3	10.7	(61.8)
Hawaii	21.3	16.2	(75.9)	19.3	15.0	(77.7)	23.2	17.3	(74.6)	20.6	16.5	(80.0)	—	—	—
Idaho	19.4	14.2	(73.3)	18.3	13.1	(71.5)	20.3	15.1	(74.6)	19.3	14.2	(73.4)	—	—	—
Illinois	19.1	13.5	(70.8)	17.6	12.6	(71.5)	20.3	14.3	(70.5)	19.2	13.8	(72.1)	17.7	11.4	(64.5)
Indiana	18.5	13.2	(71.5)	17.0	12.2	(71.6)	19.7	14.1	(71.5)	18.5	13.4	(72.4)	17.7	10.4	(59.1)
Iowa	19.4	14.8	(76.4)	17.8	13.4	(75.3)	20.7	15.9	(77.1)	19.4	14.9	(76.7)	17.4	7.1	(40.9)
Kansas	19.0	14.2	(75.0)	17.6	13.1	(74.8)	20.1	15.1	(75.2)	19.0	14.4	(75.6)	17.4	11.2	(64.1)
Kentucky	17.6	11.0	(62.1)	16.2	10.2	(63.2)	18.9	11.6	(61.5)	17.7	12.2	(68.9)	16.8	9.4	(56.2)
Louisiana	17.9	12.0	(67.1)	16.4	11.6	(70.4)	19.0	12.3	(64.8)	18.1	12.7	(70.4)	16.9	9.2	(54.1)
Maine	19.0	14.7	(77.3)	17.5	13.5	(77.0)	20.2	15.7	(77.5)	19.0	14.7	(77.7)	—	—	—
Maryland	19.2	14.4	(75.1)	17.8	13.3	(75.0)	20.4	15.3	(75.2)	19.3	14.8	(76.7)	18.2	12.5	(68.7)
Massachusetts	19.7	15.0	(76.1)	18.2	13.8	(76.0)	20.9	15.9	(76.2)	19.6	15.1	(77.4)	21.1	12.8	(60.7)
Michigan	18.8	13.9	(73.6)	17.4	12.8	(73.7)	20.0	14.7	(73.6)	18.9	14.3	(75.3)	17.6	10.6	(60.0)
Minnesota	20.1	15.6	(77.5)	18.6	14.3	(76.7)	21.4	16.7	(78.2)	20.2	15.6	(77.4)	20.2	11.5	(56.9)
Mississippi	17.5	10.8	(61.5)	15.8	10.1	(63.9)	19.0	11.4	(59.9)	17.9	11.8	(66.0)	16.6	8.2	(49.2)
Missouri	18.5	13.0	(70.2)	17.1	11.7	(68.8)	19.6	14.0	(71.1)	18.5	13.2	(71.3)	17.4	10.0	(57.3)
Montana	19.2	14.6	(75.9)	18.1	13.4	(74.1)	20.2	15.6	(77.2)	19.3	14.7	(76.4)	—	—	—
Nebraska	19.3	14.5	(75.3)	17.9	13.1	(73.1)	20.4	15.7	(76.7)	19.3	14.7	(75.9)	17.3	9.1	(52.4)
Nevada	18.7	13.7	(73.2)	17.4	12.8	(73.3)	19.9	14.5	(73.1)	18.4	13.6	(74.2)	18.8	14.0	(74.5)
New Hampshire	19.4	15.1	(77.8)	18.0	14.0	(77.5)	20.6	16.0	(77.9)	19.3	15.1	(77.9)	—	—	—
New Jersey	19.6	14.0	(71.2)	18.1	12.9	(71.4)	20.8	14.8	(70.9)	19.6	14.4	(73.5)	18.5	10.8	(58.6)
New Mexico	19.6	13.9	(71.0)	18.4	13.1	(71.2)	20.8	14.7	(70.8)	19.6	14.3	(72.8)	19.8	15.1	(76.5)
New York	20.0	14.5	(72.5)	18.5	13.6	(73.5)	21.2	15.3	(71.9)	19.8	14.8	(75.0)	20.6	12.2	(59.0)
North Carolina	18.6	12.7	(68.3)	17.1	11.7	(68.5)	19.8	13.5	(68.1)	18.8	13.3	(70.6)	17.6	10.1	(57.2)
North Dakota	19.9	14.6	(73.5)	18.1	12.9	(71.6)	21.5	16.0	(74.6)	20.0	14.8	(74.0)	—	—	—
Ohio	18.5	13.2	(71.4)	17.0	12.2	(71.5)	19.6	14.0	(71.4)	18.5	13.4	(72.5)	17.4	9.7	(55.6)
Oklahoma	17.7	12.0	(67.6)	16.4	10.8	(66.0)	18.8	12.9	(68.7)	17.8	12.3	(69.2)	16.7	9.9	(59.3)
Oregon	19.3	15.0	(77.9)	18.0	13.9	(77.3)	20.4	16.0	(78.3)	19.3	15.0	(78.1)	18.9	12.4	(65.6)
Pennsylvania	18.9	13.9	(73.6)	17.4	12.8	(73.8)	20.2	14.8	(73.5)	19.0	14.1	(74.4)	17.6	11.5	(65.2)
Rhode Island	19.4	14.5	(74.7)	17.8	13.1	(73.7)	20.7	15.6	(75.3)	19.4	14.6	(75.2)	21.9	14.6	(66.5)
South Carolina	18.5	12.9	(69.7)	16.9	12.0	(70.6)	19.7	13.6	(69.0)	18.8	13.7	(73.2)	17.2	9.8	(57.0)
South Dakota	19.8	15.0	(75.6)	18.0	13.3	(74.0)	21.3	16.4	(76.7)	20.0	15.2	(76.0)	—	—	—
Tennessee	18.0	11.9	(66.2)	16.5	11.2	(68.0)	19.2	12.5	(65.1)	18.1	12.0	(66.5)	16.6	10.3	(62.3)
Texas	18.9	12.9	(68.4)	17.5	12.2	(69.8)	20.0	13.4	(67.3)	18.9	13.6	(72.1)	17.2	10.6	(61.9)
Utah	19.8	15.0	(75.7)	18.9	14.3	(75.7)	20.7	15.7	(75.9)	19.8	15.0	(76.0)	—	—	—
Vermont	19.4	15.2	(78.2)	18.0	13.9	(77.4)	20.6	16.2	(78.8)	19.4	15.2	(78.5)	—	—	—
Virginia	18.9	14.2	(74.9)	17.5	13.3	(76.2)	20.1	14.9	(74.0)	19.1	14.4	(75.7)	17.5	11.7	(66.9)
Washington	19.4	15.1	(77.6)	18.1	14.0	(77.3)	20.5	16.0	(77.9)	19.3	15.1	(78.2)	18.7	11.0	(58.9)
West Virginia	17.5	11.0	(63.0)	16.2	10.3	(64.1)	18.6	11.6	(62.3)	17.5	11.0	(63.1)	16.8	10.0	(59.8)
Wisconsin	19.5	14.9	(76.4)	18.0	13.4	(74.7)	20.8	16.1	(77.6)	19.6	15.0	(76.7)	17.6	12.2	(69.5)
Wyoming	19.0	14.4	(75.8)	17.9	13.7	(76.3)	20.1	15.2	(75.5)	19.1	14.5	(76.2)	—	—	—
**State average** **	**19.1**	**13.9**	**(72.7)**	**17.7**	**12.9**	**(72.7)**	**20.3**	**14.8**	**(72.7)**	**19.2**	**14.3**	**(74.5)**	**18.1**	**11.1**	**(61.0)**

**Sources:** CDC, National Vital Statistics System, Behavioral Risk Factor Survey System, and the U.S. Census Bureau.

* Calculated from LE and HLE with multiple decimal places.

† All pairwise comparisons for HLE between males and females were significantly different in all states, at p<0.05 based on two-tailed z-statistics.

§ In all states for which there were sufficient data, all differences between HLE for blacks and whites were significantly different at p<0.05 based on two-tailed z-statistics, except for Nevada, New Mexico, Rhode Island, and West Virginia.

¶ States with <700 deaths in a subpopulation during 2007–2009.

** Average across state estimates.

## Footnotes

* Additional information about BRFSS is available at http://www.cdc.gov/brfss.

† The response rate reflects telephone sampling efficiency and the degree of participation among eligible respondents contacted. The cooperation rate reflects the proportion of persons who completed an interview among eligible persons contacted.

§ Methodologies used for calculating HLE are available at http://www.ncbi.nlm.nih.gov/pmc/articles/PMC1937122 and http://www.cdc.gov/nchs/data/statnt/statnt21.pdf.
